# Performance and mechanism of removal of antibiotics and antibiotic resistance genes from wastewater by electrochemical carbon nanotube membranes

**DOI:** 10.3389/fchem.2022.973490

**Published:** 2022-08-08

**Authors:** Jun Wang, Hong Liu, Xiaofei Chen, Ye Li, Xueni Sha, Huanjie Song, Bolin Li, Zheng Yan, Ming Chang

**Affiliations:** ^1^ School of Resource and Environmental Engineering, Wuhan University of Technology, Wuhan, China; ^2^ Wuhan Lingang Economic and Technological Development Zone Service Industry Development Investment Group Co. LTD, Wuhan, China; ^3^ Hubei Academy of Ecological Environment Sciences, Wuhan, China; ^4^ Chinese Society for Environmental Sciences, Beijing, China; ^5^ State Key Laboratory of Environmental Criteria and Risk Assessment, Chinese Research Academy of Environmental Science, Beijing, China

**Keywords:** carbon nanotubes, antibiotics, antibiotic resistance genes, removal mechanism, electrochemical oxidation

## Abstract

Electrochemical carbon nanotube (CNT) and carboxylated carbon nanotube (CNT-COOH) membranes were prepared by vacuum filtration for the removal of antibiotics and antibiotic resistance genes (ARGs) from water. Scanning electron microscopy and energy-dispersive spectroscopy were used to analyze the performances of the two electrochemical membranes in the removal of antibiotics and ARGs, to determine the effects of different factors on removal rates, and to explore the mechanisms of the removal of antibiotics and ARGs. The results showed that CNT-COOH formed a porous mesh structure on the surface of polytetrafluoroethylene membrane and contained more oxygen than CNT. The electrochemical CNT-COOH membrane showed higher antibiotic and ARG removal rates than the electrochemical CNT membrane, with an antibiotics removal rate of 82% after 60 min of reaction and an ARGs concentration decrease by 1.85 log. The removal rate of antibiotics and ARGs increased with the increase in electrolyte concentration and anode voltage but decreased with the increase in the influent flow rate. The removal rate of antibiotics decreased with the increase in pH, while the best removal rates of ARGs were observed in a neutral environment. The degradation mechanism of antibiotics on the electrochemical CNT-COOH membrane was analyzed, and possible antibiotic degradation pathways were proposed. The removal of antibiotics and ARGs mainly occurred through electrochemical degradation, where hydroxyl radicals (-OH) played a dominant role.

## Introduction

Antibiotics are compounds used to treat microbial infectious diseases and have been widely used in the treatment of human and animal diseases, as well as in aquaculture and livestock farming (Manzetti and Ghisi, 2014). The misuse of antibiotics has recently raised public concern. Moreover, during the production and application of antibiotics, large amounts of antibiotic-containing wastewater are produced and discharged into the environment, resulting in serious pollution (Focazio et al., 2008). The residual antibiotics are persistent and difficult to degrade by conventional treatment methods (Kümmerer et al., 2000; Kümmerer, 2009a; Wang and Wang, 2016). Therefore, antibiotics have often been detected in various natural environments (Wang and Wang, 2019) including river water (Huang et al., 2019), groundwater (Szekeres et al., 2018), surface water (Danner et al., 2019), soil (Cerqueira et al., 2019), sediment (Chen and Zhou, 2014), and drinking water (Sanganyado and Gwenzi, 2019). The long-term presence of antibiotics in the natural environment may lead to the generation of antibiotic resistance genes (ARGs) (Dantas et al., 2008) and antibiotic-resistant bacteria (ARB), accelerating the spread of antibiotic resistance, which poses threat to human health and ecosystems (Kümmerer, 2009b).

Membrane technology is considered one of the most promising water treatment methods because of its high separation selectivity, low energy consumption, lack of requirement for additional chemicals, relatively easy scaling, and possibility of continuous operation (Ying et al., 2017; Goh and Ismail, 2018). However, the membrane process also possesses certain drawbacks. During the filtration, the accumulation of contaminants on the membrane surface and inside the membrane pores may lead to membrane fouling through blocking and rejection of the membrane (Miller et al., 2017). Thus, the prolonged use of the membrane may lead to a decrease in its performance (Jhaveri and Murthy, 2016). In addition, membrane separation is a physical process, so although contaminants such as antibiotics may be concentrated, they are not actually broken down, making it difficult to completely remove the contaminants. Electrochemical advanced oxidation process (EAOP) is also an emerging technology that can break down large organic compounds into small molecules that are easily degraded or even mineralize them into CO_2_, H_2_O or other inorganic substances. EAOP has attracted increasing attention because of its chemical-free nature, ease of control, stable performance, and environment-friendliness (Chaplin, 2014; Sirés et al., 2014; Martínez-Huitle et al., 2015; Moreira et al., 2017). However, the efficiency of conventional EAOP reactors is usually limited by the weak mass transfer of molecules in the reactor, and EAOP alone is not suitable for the treatment of low-concentration wastewater on a large scale (Ganiyu et al., 2015).

The combination of membrane technology with EAOP may alleviate the membrane contamination issue and resolve the weak mass transfer of EAOP, thus improving the overall separation performance (Yang et al., 2011; Ganiyu et al., 2015; Martínez-Huitle et al., 2015; Li et al., 2018). On the one hand, membrane filtration may be used to concentrate contaminants to the level suitable for EAOP treatment. On the other hand, it confines or retains toxic and hazardous contaminant intermediates in the membrane to degrade or remove the contaminants through reaction with oxidants such as reactive radicals (Molinari et al., 2006).

Membrane filtration is an effective method to remove contaminants from water, and there have been several studies on the removal of micropollutants from water by carbon nanotube (CNT) filtration membranes. Wang et al. (Wang et al., 2018) investigated the removal of acetaminophen, triclosan, caffeine, and carbendazim from water by CNT membrane filtration. Sun et al. (Sun et al., 2020) investigated the removal of tetracycline antibiotics from water by mixed matrix carbon membranes. Wang et al. (Wang et al., 2015) used nanocomposite membranes containing single-walled and multi-walled CNTs to remove triclosan and ibuprofen. Wu et al. (Wu et al., 2016) removed bisphenol A and norfloxacin from drinking water using acid-treated CNTs and polyvinyl chloride ultrafiltration membranes. Guo et al. designed a silicate-enhanced fluidized electro-Fenton system with a nanoconfined catalyst for the removal of tetracycline (Guo et al., 2021). Zheng et al. designed an electrocatalytic filtration system with carbon nanotube supported nanoscale zerovalent copper toward ultrafast oxidation of organic pollutants (Zheng et al., 2021).

In this study, electrochemical CNT and carboxylated CNT (CNT-COOH) membranes for the removal of antibiotics and antibiotic resistance genes (ARGs) from water were prepared by vacuum filtration. The two electrochemical membranes were studied using scanning electron microscopy (SEM) and energy-dispersive spectroscopy (EDS) to explore the effectiveness of the removal of antibiotics and ARGs and factors affecting it. The intermediate products in the degradation of antibiotics were also investigated to explore the mechanism of the removal of antibiotics and ARGs.

## Materials and methods

### Chemicals and materials

Sodium Sulfate (purity ≥99.5%, analytical grade) was purchased from Sinopharm Chemical Reagent Co. Ltd. The four antibiotics were: sulfamethoxazole (SMX, purity ≥98%, C_10_H_11_N_3_O_3_S), ofloxacin (OFL, purity ≥98%, C_18_H_20_FN_3_O_4_), tetracycline (TC, purity ≥98%, C_22_H_24_N_2_O_8_), and roxithromycin (purity ≥98%, C_41_H_76_N_2_O_15_), which were purchased from Shanghai Maikelin Biochemical Technology Co. Ltd. The four ARG standards (*sul1, tetO, ermA, qnrD*) and their primers were purchased from Shanghai Wcgene Biotechnology Co. Ltd. See Supplementary Table S1 for primer names, sequences, corresponding lengths of amplified fragments, and annealing temperatures. Nanotubes and CNT-COOH (w ≥ 95%) were purchased from Chengdu Organic Chemistry Co., Ltd. Chinese Academy of Sciences.

### Preparation of electrochemical CNT membranes

**FIGURE 1:**
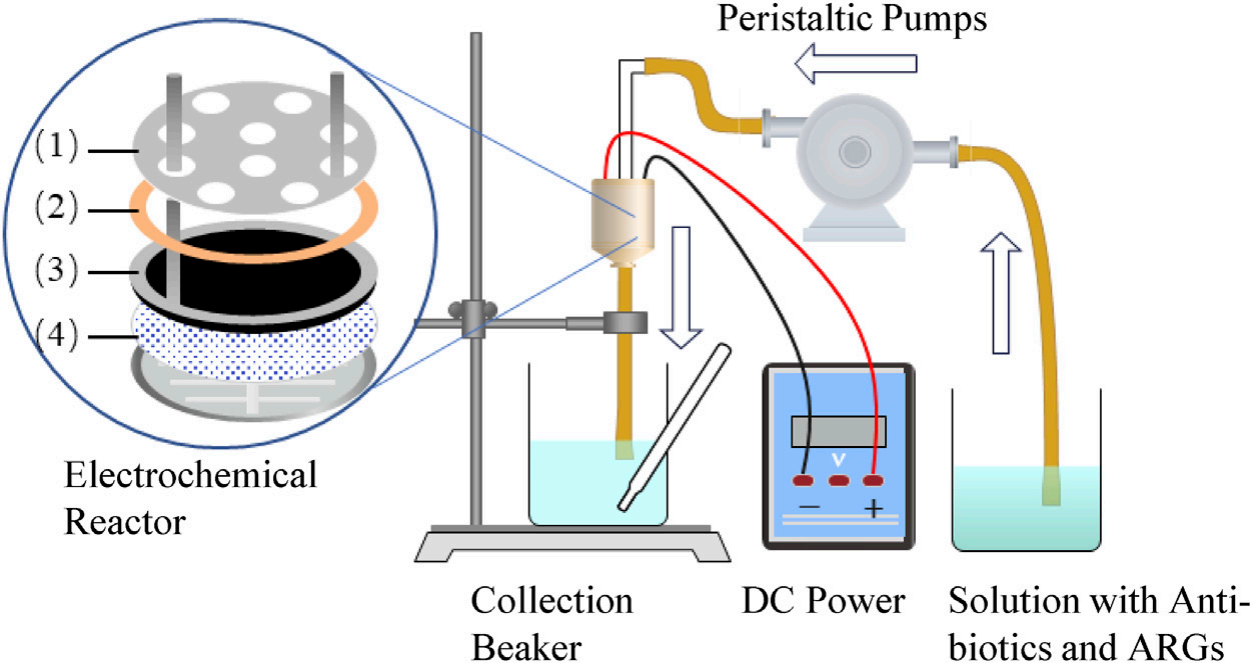
Schematic of the experimental setup. The electrochemical membrane reactor was designed with a polycarbonate filter casing, which included (1) a perforated titanium cathode, (2) an insulated silicone electrode spacer and seal (0.8 mm), (3) a titanium anode ring pressed onto a CNT anode, and (4) a CNTM/CNTM-COOH anode reactor supported by a PTFE membrane.

CNT and CNT-COOH powders (25 mg) were separately dispersed in low-concentration solutions of surfactant dimethylamide. The prepared CNT and CNT-COOH solutions were sonicated for 30 min and then filtered separately under vacuum at a pressure of 5 KPa. A polytetrafluoroethylene (PTFE) filter membrane with a pore size of 5 μm was used as the substrate. After the filtration was completed, the filter membrane was gently removed from the filtration device, flattened with the pressure of a heavy object, and dried in the air. The dried CNT membrane (CNTM) and CNT-COOH membrane (CNTM-COOH) were then dried in an oven at a constant 100 °C temperature for 2 h; after cooling, they were stored in a cool and dry place for further use. The structural composition of the electrochemical membrane reactor was shown in Figure 1.

### Characterization methods

The surface morphology of electrochemical CNT membranes was observed using SEM (SIGMA HD field emission scanning electron microscope, ZEISS, Germany). Elemental microanalysis was performed using EDS (X-Max N80, ZEISS, Germany). The samples were plated with copper prior to qualitative and semi-quantitative elemental analysis of the test locations.

### Study on the electrochemical filtration performance of CNT membranes

Antibiotic mixture solution with a concentration of 0.25 mg/L and ARGs mixture solution of 1 × 10^9^ copies/mL were prepared. The differences in the removal performance of the electrochemical CNTM and CNTM-COOH were evaluated by using them as anodes of the electrochemical membrane reactor.

The effects of the following experimental parameters: electrolyte (Na_2_SO_4_) concentration (0.001, 0.01, and 0.1 mol/L), voltage (0, 1.0, 2.0, and 3.0 V), flow rate (5, 10, and 15 ml/min), and pH (3, 5, 7, 9, and 11) on the filtration performance were investigated.

Wastewater containing antibiotics and ARGs was fed from the inlet container through a peristaltic pump to the water inlet at the upper end of the reactor casing and flowed out the lower outlet after passing through the electrochemical CNT membrane reactor. Water samples were taken periodically at the outlet to determine the concentration of antibiotics and ARGs in the effluent.

### Analysis methods

Antibiotics were detected using high-performance liquid chromatography (HPLC). The analytical column used for antibiotic detection was ACQUITY UPLC BEH C18 (2.1 × 100 mm, l.7 μm; Waters, United States). The mobile phases were 0.1% formic acid in ultrapure water (v/v) and 0.1% formic acid in acetonitrile. The sample injection volume was set to 5 μL, and the gradient elution mode was selected, and the elution procedure is shown in Supplementary Table S2. Determination of intermediate products of antibiotics degradation by high performance liquid chromatography tandem quadrupole time-of-flight mass spectrometry (Q-ToF-MS). Mass spectrometry was performed using an electrospray ionization source in positive ion mode (ESI+) and multiple reaction detection mode. The capillary voltage was 3.0 kV, the cone voltage was set to 6–100 V, and the ion source temperature was set to 150°C. The temperature of the desolvation gas (N_2_) was set to 550°C, and the flow rate of the desolvation gas was 1000 L/h. The flow rate of the cone gas (N_2_) was 50 L/h, the flow rate of the impingement gas (Ar) was set to 0.15 ml/min, and the column temperature was 40°C.

DNA was extracted using the PowerSoil DNA Isolation kit, and the extracted DNA samples were stored at -20°C until subsequent gene quantification assays. PCR amplification, as well as quantification of ARGs, was performed by using quantitative PRC (qPCR). qPCR was performed using a fluorescent quantitative PCR instrument (qTower2.2, Jena, Germany).

The removal rate of antibiotics was calculated using Equation 1.

$$R\left(\%\right)=(C_{(in)}-C_{(out)})/C_{(in)}$$ (1)

The removal rate of ARGs was calculated using Equation 2.

$$R_{ARGs}=Log\;C_{\left(in\right)}-Log\;C_{(out)}$$ (2)

where *R*_*ARGs*_ is the logarithm (log) of the removal rate of ARGs by the reactor. *C*_*(in)*_ and *C*_*(out)*_ are the pollutant concentrations in the influent and effluent, respectively.

## Results and discussion

### Characterization of electrochemical CNT membranes

**FIGURE 2:**
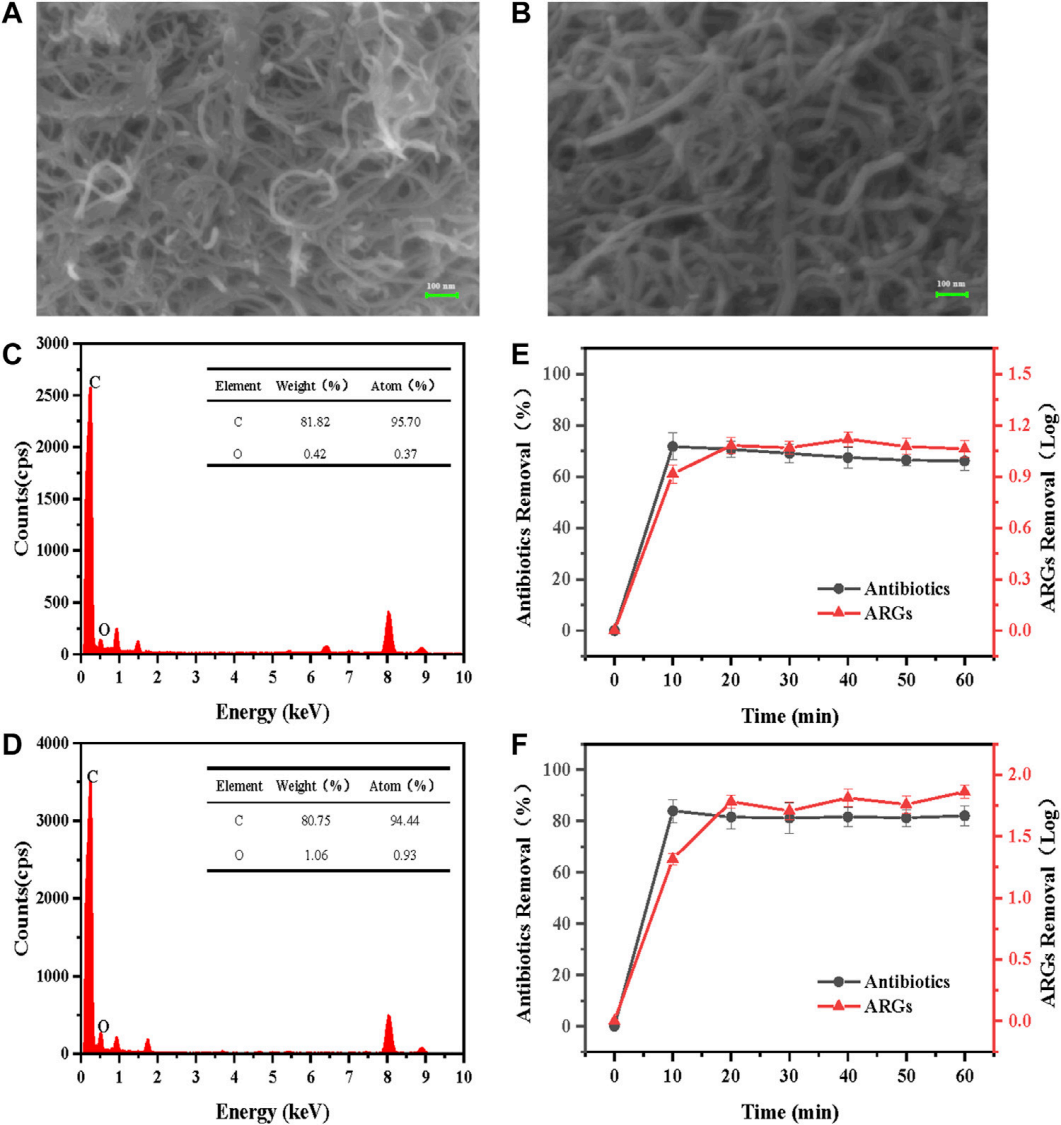
**(A)**–**(B)** SEM and **(C)**–**(D)** EDS characterization images and **(E)**–**(F)** performance of CNTM and CNTM-COOH.

It can be seen from Figure 2A that the conventional CNTs are entangled and agglomerated in lumps, which could not be well dispersed even after ultrasonication. Compared with CNT, CNT-COOH is less prone to agglomeration better dispersion, its surface is smoother, the truncated surface is more obvious, and it can maintain a larger dispersion. From Figure 2B, the surface of CNTM-COOH is clean and more uniform than that of CNTM, and the three-dimensional network structure formed by individual CNTs and the intertwining of CNTs could be clearly observed, with gaps of different sizes between individual nanotubes. This indicated that after vacuum filtration, CNT-COOH covered the surface of the PTFE membrane uniformly and formed a porous mesh structure on the surface of the PTFE membrane. In addition, it has been shown that CNT can produce more defective sites and polar groups, such as carboxyl groups, after acidification, and it is these introduced oxygen-containing groups that improve its dispersibility.

The contents of C and O in the two different CNT types are shown in Figure 2C below. The content of C in CNT was 95.70%, and that of O was 0.37%; From Figure 2D, the weight percentages of C and O in CNT-COOH were 94.44% and 0.93%, respectively, which showed that CNT-COOH contained more oxygen-containing groups. This may be attributed to the introduction of O on the surface of CNT-COOH by the strong acid during carboxylation.

### Comparison of the removal performance of electrochemical CNT membranes

The anode voltage was set to 1 V, the flow rate was set to 10 ml/min, and the concentration of electrolyte Na_2_SO_4_ was set to 0.01 mol/L to treat antibiotics and ARGs with concentrations of 0.25 mg/L and 1 × 10^9^ copies/mL The results are shown in Figures 2E,F.

Since the reactor is operated in a vertical flow mode, the influent concentration is constant (without considering the hydrolysis of antibiotics and ARGs) and the effluent water is not returned to the reactor, so the effluent concentration should remain stable after a period of reactor operation due to the continuous oxidation reaction on the carbon nanotube membrane electrodes to degrade pollutants. The removal of antibiotics by the electrochemical CNTM reactor was approximately 72% after 60 min of operation, and the concentration of ARGs decreased by 1.1 log. After 60 min of operation of the electrochemical CNTM-COOH reactor, the removal of antibiotics by the reactor was as high as 82%, and the ARG concentration decreased by approximately 1.85 log, with stable removal capacity and a removal efficiency higher than that of CNTM. This may be attributed to the better dispersion of CNTM-COOH after carboxylation and higher oxygen-containing group content of CNTM-COOH. In addition, more defects and active sites were created during the acidification (Gao et al., 2015), which gave CNTM-COOH higher oxygen evolution potential and therefore better electrochemical oxidation properties.

### Effect of electrolyte concentration

**FIGURE 3:**
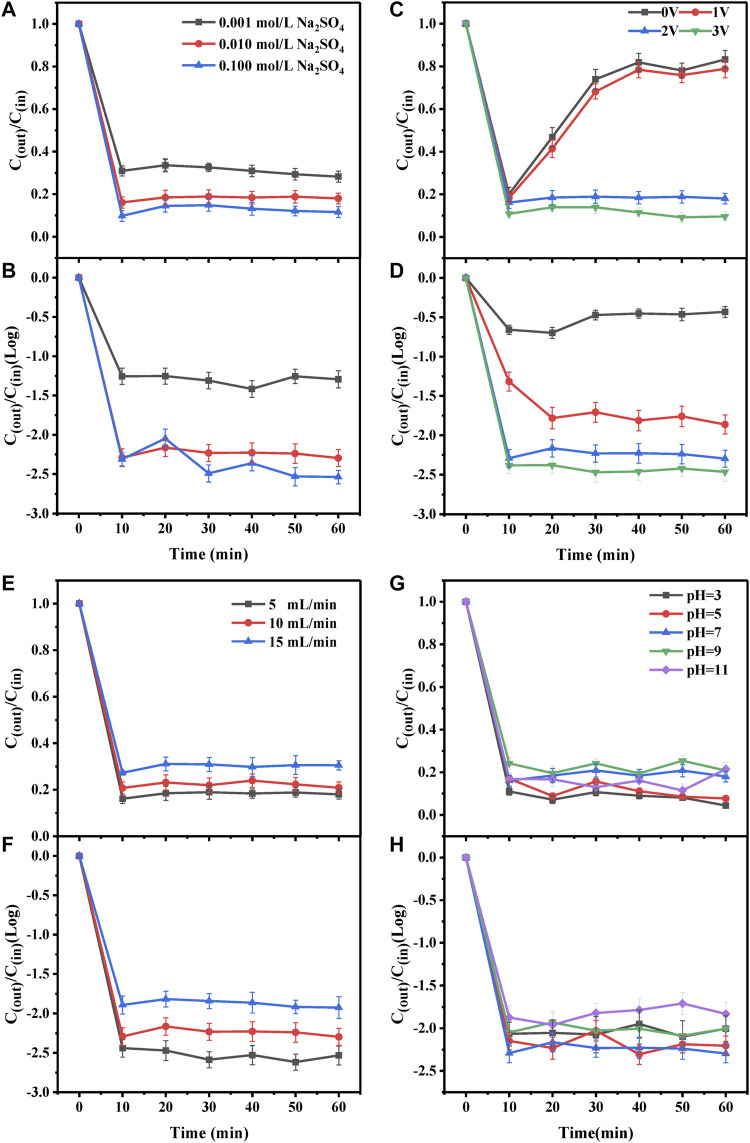
Effect of **(A)**–**(B)** electrolyte concentration **(C)**–**(D)** anode voltage **(E)**–**(F)** flow rate and **(G)**–**(H)** pH on the degradation of antibiotics and ARGs by the electrochemical CNTM-COOH reactor.

The removal of the antibiotics and ARGs by the electrochemical CNT membrane reactor at different concentrations (0.001, 0.01, and 0.1 mol/L) of electrolyte (Na_2_SO_4_) are shown in Figures 3A,B. With the increase in Na_2_SO_4_ concentration, the concentrations of antibiotics and ARGs in the reactor effluent gradually decreased, which was attributed to the increased electrolysis efficiency because with increasing electrolyte concentration, the conductivity of the reaction system also increased. However, the effect of Na_2_SO_4_ concentration on the oxidative degradation of antibiotics and ARGs was limited. When the Na_2_SO_4_ concentration was increased from 0.01 to 0.1 mol/L, the changes in the concentrations of antibiotics and ARGs in the effluent were not significant despite a 10-fold increase in electrolyte concentration. This may be explained by the migration of SO_4_^2-^ to the anode to occupy the active site on the anode at high Na_2_SO_4_ concentrations, thus reducing the removal rate. Yang et al. (Yang et al., 2020) found in the study on the degradation of 4-epi-anhydrochlotetracycline that excessive SO_4_^2-^ occupied the active sites on the electrode. Chen et al. (Chen et al., 2015) and Zhong et al. (Zhong et al., 2013) also reported that at high Na_2_SO_4_ concentrations, more SO_4_^2-^ migrated to the anode to occupy the active sites on the anode, thus reducing the removal efficiency.

### Effect of anode voltage

The removal rates of antibiotics and ARGs by the electrochemical CNT membrane reactor at different voltages (0, 1.0, 2.0, and 3.0 V) are shown in Figures 3C,D. It can be seen from Figure 3C that the removal rate of antibiotics at 1 V is almost the same as that at 0 V, while at 2 and 3 V, the removal rates of antibiotics reach 80 and 90%, respectively. The slight increase in the removal effect at 1 V compared to 0 V may be explained by electrostatic adsorption (Sun et al., 2020). Figure 3D shows that the concentration of ARGs only decreased by approximately 0.4–0.5 log at a voltage of 0 V. ARGs may be removed mainly through adsorption. However, the concentration decreased by 2.2–2.3 log at voltages of 2 and 3 V. Significant degradation of antibiotics and ARGs at 2 and 3 V may be attributed to the increase in the number of electrons generated with the increase in voltage, which increased the content of hydroxyl radicals as well as improved the removal of pollutants (Liu and Vecitis, 2012). However, too high anode potential may also lead to water-resolving oxygen reactions, and there is direct competition between organic oxidation and oxygen precipitation reactions. In addition, the high anode potential may damage the structure of carbon nanotubes, which is not conducive to the normal and stable operation of the carbon nanotube electrochemical membrane reactor. Therefore, the optimal anode voltage was chosen to be 2.0 V for the experiment.

### Effect of inlet water flow

The removal rates of antibiotics and ARGs by the electrochemical CNT membrane reactor at different flow rates (5, 10, and 15 ml/min) are shown in Figures 3E,F. The removal rates for antibiotics and ARGs decreased with the increase in the flow rate. The lowest antibiotic concentration in the effluent was observed at the flow rate of 5 ml/min. This may be explained by the low flow rate resulting in an increase in retention time, which significantly prolonged the degradation time of different pollutants and their intermediates, resulting in their fuller degradation (Cui et al., 2009). However, the volume of antibiotic wastewater treated per unit time was also lower at lower flow rates. In a study by Liu et al. (Liu et al., 2016), the efficiency of antibiotics removal by electrochemical membranes increased with the decrease in flow rate. The lower the flow rate, the longer the retention time, and the more complete the degradation of TC. This was attributed to the longer duration of the contact between the pollutants and electrodes causing the reaction to proceed to greater completion. However, when the influent flow rate was too small and the retention time was too long, especially when exceeding 4.4 min, the degradation of TC by the electrochemical membrane became less effective. This was because the liquid mass transfer efficiency in the reactor became low owing to the prolonged retention time.

### Effect of solution pH

The removal of antibiotics and ARGs by the electrochemical CNT membrane reactor at different pH (pH = 3, 5, 7, 9, and 11) is shown in Figures 3G,H. Figure 3G shows that the maximum removal of antibiotics was 95.6% at a pH of 3, and the lowest removal of antibiotics was 75.5% at a pH of 11. The different removal rates at different pH values may be attributed to the fact that antibiotics have different morphology at different pH levels (Gao et al., 2014), thereby affecting the degradation mechanism and the removal rate (Fan et al., 2016). On the one hand, acidic conditions may have accelerated the hydrolysis of antibiotic molecules in the aqueous solution (Sopaj et al., 2020). On the other hand, H^+^ in acidic conditions facilitated the production of -OH, strong oxidizing properties of which played an important role in the degradation of antibiotics, and that H_2_O_2_ decomposes to O_2_ under basic conditions (Liu et al., 2020). Therefore, the removal rate of antibiotics under acidic conditions was higher. Figure 3H shows that the removal rate of ARGs increased with increasing pH in the range of 1–7 but decreased with increasing pH above 7. The concentration of ARGs decreased by 2.30 log at a pH of 7. At the pH below 7, the phosphate groups on ARGs tended to be positively charged by protonation, and the increase in surface charge led to repulsion between the positively charged CNTM-COOH (pH = 1–3) and the protonated phosphate groups in ARGs (Vecitis et al., 2011), which led to a decrease in the ARGs removal rate. At the pH exceeding 7, ARGs became deprotonated and negatively charged, and interacted with the cations in the solution (e.g., Na^+^ ions), which disrupted the structure and surface charge of ARGs. This affected the interaction between ARGs and the electrochemical CNT membrane and reduced the mass transfer, resulting in a decrease in the removal rate (Vecitis et al., 2011).

### Intermediate products and degradation pathways

Liquid chromatography coupled with mass spectrometry was used to further identify the intermediate products to elucidate the effects of -OH, which was generated during the electrochemical process, on the oxidative degradation of antibiotics. OFL and SMX were selected as study objects to propose reasonable speculations on the degradation pathways of antibiotics based on the intermediates detected during degradation based on the results of previous studies. The structural information for OFL and SMX is shown in Supplementary Figures S1, S2.

The intermediates of OFL during the electrochemical degradation were identified using HPLC-Q-TOF-MS/MS, and four major intermediates with mass-to-charge ratios (m/z) of 378, 336, 348, and 294 were ultimately detected. The molecular structures and other information on these four compounds are provided in Supplementary Table S5, and the mass spectral data are shown in Supplementary Figures S3, S4.

The substituent of the piperazine ring of OFL was changed in the electrochemical oxidative degradation system, in which the piperazine ring was first hydroxylated to generate product P378 (m/z = 378), which was subsequently dehydrated to generate product P360 (m/z = 360). The methyl group on the piperazine ring was removed to generate product P348 (m/z = 348). Both the piperazine and oxazine rings of OFL were opened to generate product S294 (m/z = 294) (Gong et al., 2016), and the removal of the ethyl group at N1 or the methyl group at N4 on the piperazine ring produced P336 (m/z = 336).

**FIGURE 4:**
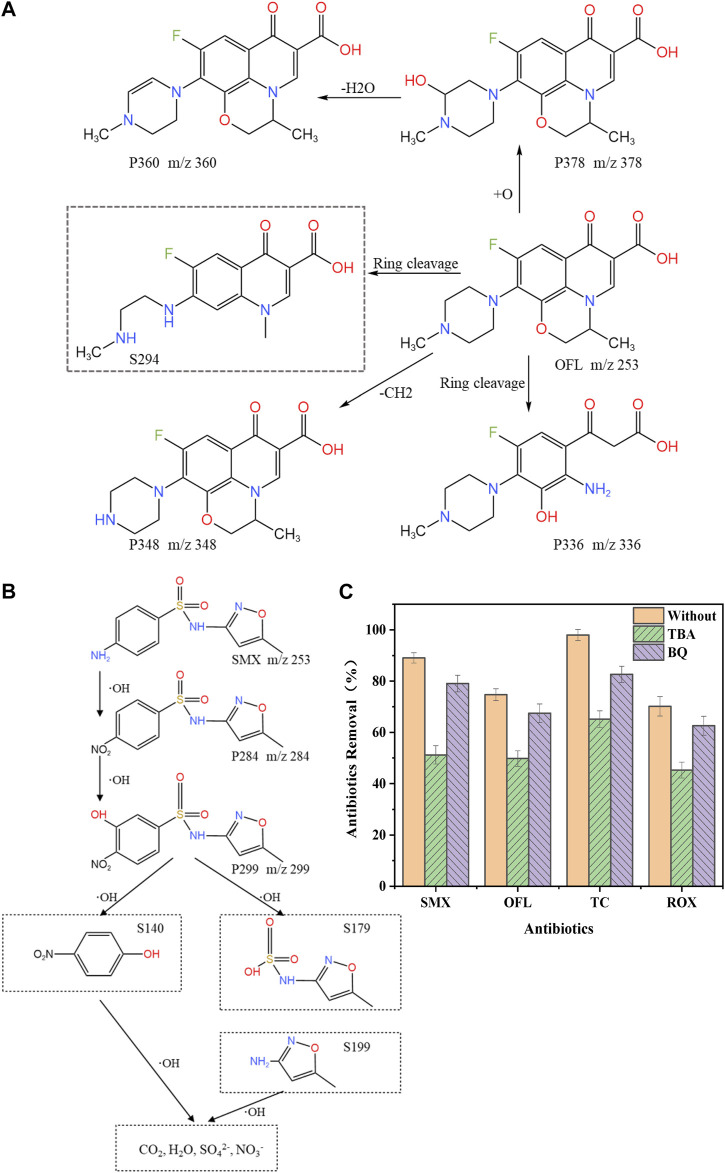
Proposed degradation pathway and mechanisms of antibiotics. **(A)** OFL **(B)** SMX **(C)** Influence of TBA and BQ on the electrochemical oxidation of antibiotics.

Based on the results of the intermediate product analysis, the proposed degradation pathway of OFL is shown in Figure 4A. The degradation of OFL consisted of two oxidation pathways, namely the demethylation reaction and the hydroxylation and decarboxylation of piperazine. Pathway I: the piperazine ring of OFL was attacked by hydroxyl radicals to produce a mono-hydroxyl derivative, namely the degradation product P378, in which a water molecule was then removed to generate product P360. Pathway II: degradation product S294 was generated through the removal of the ethyl group at N1 or the methyl group at N4 on the piperazine ring of the OFL molecule, followed by decarboxylation to remove methyl and carboxyl groups. Ultimately, the intermediate products mentioned above underwent oxidation to produce CO_2_ and H_2_O (Wang et al., 2022).

The intermediates of SMX during electrochemical degradation were identified using the HPLC-Q-TOF-MS/MS, and two major intermediate products with mass-to-charge ratios (m/z) of 284 and 299 were detected. The information such as molecular structures are provided in Supplementary Table S6, and the MS data for these two compounds are shown in Supplementary Figure S5.

Based on the results of the analysis of intermediates, the tentative degradation pathway of SMX is shown in Figure 4B. The actual detected compounds were labeled as “P”, while other intermediates postulated based on previous studies (Wang and Wang, 2018; Yin et al., 2019; Wang et al., 2020) were noted with dashed rectangles and labeled as “S". The degradation was presumed to be mainly occur through the oxidation of the phenylamine group into nitro-SMX derivatives, and similar results were obtained by Wang et al. (Wang and Wang, 2018).

In the above pathway, the phenylamine group is oxidized by -OH to produce nitro-SMX derivatives. This may be attributed to the fact that the rapid hydration of N-centered radicals formed aniline radicals, which were further neutralized by oxidation, leading to the hydrolysis of hydroxylamine and nitroso derivatives to produce nitro-SMX (Wang et al., 2020). Subsequently, -OH attacked the benzene ring to generate S140 and S179, and then the S-N single bond was attacked by -OH, leading to the breakage of the sulfonamide bond and the generation of S99. Through the successive hydroxylation of these primary products and by-products, SMX may eventually be mineralized to CO_2_, H_2_O, SO_4_^2-^, and NO_3_^−^. These reaction pathways were consistent with the findings of previous studies on SMX degradation, particularly on SMX degradation using Ti/SnO_2_-Sb/Ce-PbO_2_ anode (Liu et al., 2013) and photocatalysis (Su et al., 2016).

Smaller intermediate molecules were not detected in this study, but previous studies have demonstrated the generation of small organic molecules, such as aniline, 1,4-benzoquinone, as well as several derivatives such as aromatic sulfonamides (Yin et al., 2019; Wang et al., 2020). Aliphatic intermediates produced during electrolysis were eventually mineralized into CO_2_ and H_2_O (Dirany et al., 2010; Amina et al., 2018).

### Mechanisms of electrochemical oxidative degradation

The mechanism of electrochemical oxidative degradation reaction follows a multi-step process: firstly, deposition and adsorption of antibiotics on the electrode surface, secondly, degradation and molecular transformation of antibiotics, and finally, flaking of degradation products (Tan et al., 2020). Direct oxidation is the oxidation of the antibiotic attached to the CNT electrochemical membrane reactor, which is an electronic process. Indirect oxidation has two steps, the first step is the generation of some dissolved free radicals on the anode. The second step is the stepwise oxidation of the antibiotic by the generated oxidant (Liu et al., 2020). To determine the effect of the indirect oxidation pathways on the electrochemical oxidation of antibiotics on CNT electrodes, tert-butanol (TBA) and benzoquinone (BQ) were added to the simulated aqueous solutions of antibiotics, respectively, to capture the -OH and O_2_^−^ radicals that may be produced during degradation to identify the main radical groups that affected the degradation of antibiotics. The experimental results are shown in Figure 4C. The addition of both TBA and BQ resulted in lower removal rates of antibiotics. The removal rate of antibiotics in the TBA group was lower than that in the BQ group, which indicated that -OH plays a dominant role and -O^2-^ plays a secondary role in the indirect oxidative degradation of antibiotics and ARGs on electrochemical CNT membranes.

## Conclusion

A three-dimensional network structure was observed on the surface of the electrochemical CNTM-COOH, which had more oxygen-containing groups after carboxylation treatment, making them more effective in removing the antibiotics and ARGs than conventional electrochemical CNTMs.

The removal rates of antibiotics and ARGs increased with the increase in electrolyte concentration and anode voltage but decreased with the increase in the influent flow rate. pH mainly affected the removal through influencing the state of antibiotics and ARGs. The removal rate of antibiotics decreased with the increase in pH, with the best removal effect of ARGs obtained in a neutral environment.

The hydroxylation of SMX and ring opening reactions of benzene were the main degradation pathways of SMX, and the degradation of OFL mainly occurred via the piperazine ring. In the removal of antibiotics by electrochemical CNT membranes -OH played a primary role and -O^2-^ played a secondary role.

## Data Availability

The original contributions presented in the study are included in the article/Supplementary Material, further inquiries can be directed to the corresponding authors.
