# Supplementary material for: Performance and mechanism of removal of antibiotics and antibiotic resistance genes from wastewater by electrochemical carbon nanotube membranes
Source: Front Chem. 2022 Aug 8;10:973490. doi: 10.3389/fchem.2022.973490 (PMC9393291; doi:10.3389/fchem.2022.973490)
Supplement: Supplementary file 1 [file DataSheet1.docx]

Supplementary Material

**Table S1** Basic information on the target ARGs fragment

| Target gene | Primer name | Upper and lower primer sequences  (5'-3') | Length (bp) | Temperature (℃) |
| --- | --- | --- | --- | --- |
| *sul1* | *sul1-F*  *sul1-R* | CACCGGAAACATCGCTGCA  AAGTTCCGCCGCAAGGCT | 158 | 58 |
| *tetO* | *tetO-F*  *tetO-R* | CAACATTAACGGAAAGTTTATTGTATACCA  TTGACGCTCCAAATTCATTGTATC | 104 | 60 |
| *ermA* | *ermA-F*  *ermA-R* | TTGAGAAGGGATTTGCGAAAAG  ATATCCATCTCCACCATTAATAGTAAACC | 76 | 60 |
| *qnrD* | *qnrD-F*  *qnrD-R* | GGAGCTGATTTTCGAGGG  AGAAAAATTAGCGTAACTAAGATTTGTC | 105 | 60 |

**Table S2** Liquid chromatography conditions of antibiotics measurement

| Time (min) | Flow rate (mL/min) | Aqueous phase (%) | Organic phase (%) |
| --- | --- | --- | --- |
| 0 | 0.3 | 95 | 5 |
| 5 | 0.3 | 70 | 30 |
| 11 | 0.3 | 40 | 60 |
| 13 | 0.3 | 10 | 90 |
| 16 | 0.3 | 10 | 90 |
| 16.5 | 0.3 | 95 | 5 |
| 22 | 0.3 | 95 | 5 |

The extracted DNA was amplified by qPCR using ChamQ Universal SYBR® qPCR Master Mix produced by Nanjing Novozymes. The specific qPCR reaction system is shown in **Table S3**, and the qPCR reaction procedure is shown in **Table S4**.

**Table S3** Reaction system of qPCR

| System composition | Volume |
| --- | --- |
| 2×ChamQ Universal SYBR qPCR Master Mix | 10 µL |
| Primer 1 (10µM) | 0.4 µL |
| Primer 2 (10µM) | 0.4 µL |
| Template DNA (×10) | 2 µL |
| ddH_2_ O | up to 20 µL |

**Table S4** Reaction program of qPCR

| Cycle | Reaction temperature | Time |
| --- | --- | --- |
|  | 95°C | 30s |
| 39 Cycle | 95°C | 10s |
|  | Tm | 30s |
|  | 72°C | 30s |
|  | 60–96°C | 6min |

**Table S5** Selected intermediates of the electrochemical oxidative degradation of OFL

| Name | Molecular formula | Mass-to-charge ratio | Difference from SMX |
| --- | --- | --- | --- |
| OFL | C_18_ H_20_ FN_3_ O_4_ | 362 |  |
| P378 | C_18_ H_20_ FN_3_ O_5_ | 378 | +O |
| P336 | C_16_ H_18_ FN_3_ O_4_ | 336 | -2C2H |
| P348 | C_17_ H_18_ FN_3_ O4 | 348 | -C2H |
| P360 | C_18_ H_18_ FN_3_ O_4_ | 360 | -2H |

**Table S6** Selected intermediates of the electrochemical oxidative degradation of SMX

| Name | Molecular formula | Mass-to-charge ratio | Difference from SMX |
| --- | --- | --- | --- |
| SMX | C_10_ H_11_ N_3_ O_3_ S | 254 |  |
| P284 | C_10_ H_9_ N_3_ O_5_ S | 284 | 2H→2O |
| P299 | C_10_ H_9_ N_3_ O_6_ S | 299 | + -OH |


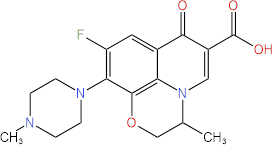


**Figure S1** Molecular structure of OFL. Ofloxacin (OFL) is a synthetic fluoroquinolone drug, which is a broad-spectrum antibiotic. Its IUPAC name is (±)-9-fluoro-2,3-dihydro-3-methyl-1o-(4-methyl-1-piperazinyl)-7-oxo-7H-pyrido[1,2,3-de]-1,4-benzoxazine-6-carboxylic acid, and the molecular formula is C_18_H_20_FN_3_O_4_. Its molecular weight is 361.14.


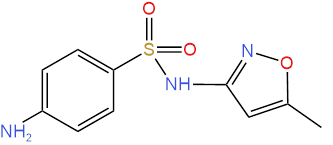


**Figure S2** Molecular structure of SMX. Sulfamethoxazole (SMX) is a broad-spectrum antimicrobial drug of the sulfonamide class. Its IUPAC name is N-(5-methyl-3-isoxazolyl)-4-aminobenzenesulfonamide, its molecular formula is C_10_H_11_O_3_N_3_S, and its molecular weight is 254.

**Figure S3** Mass spectra of OFL and m/z = 378 intermediates

**Figure S4** Mass spectra of m/z = 360, 336, 348 intermediates


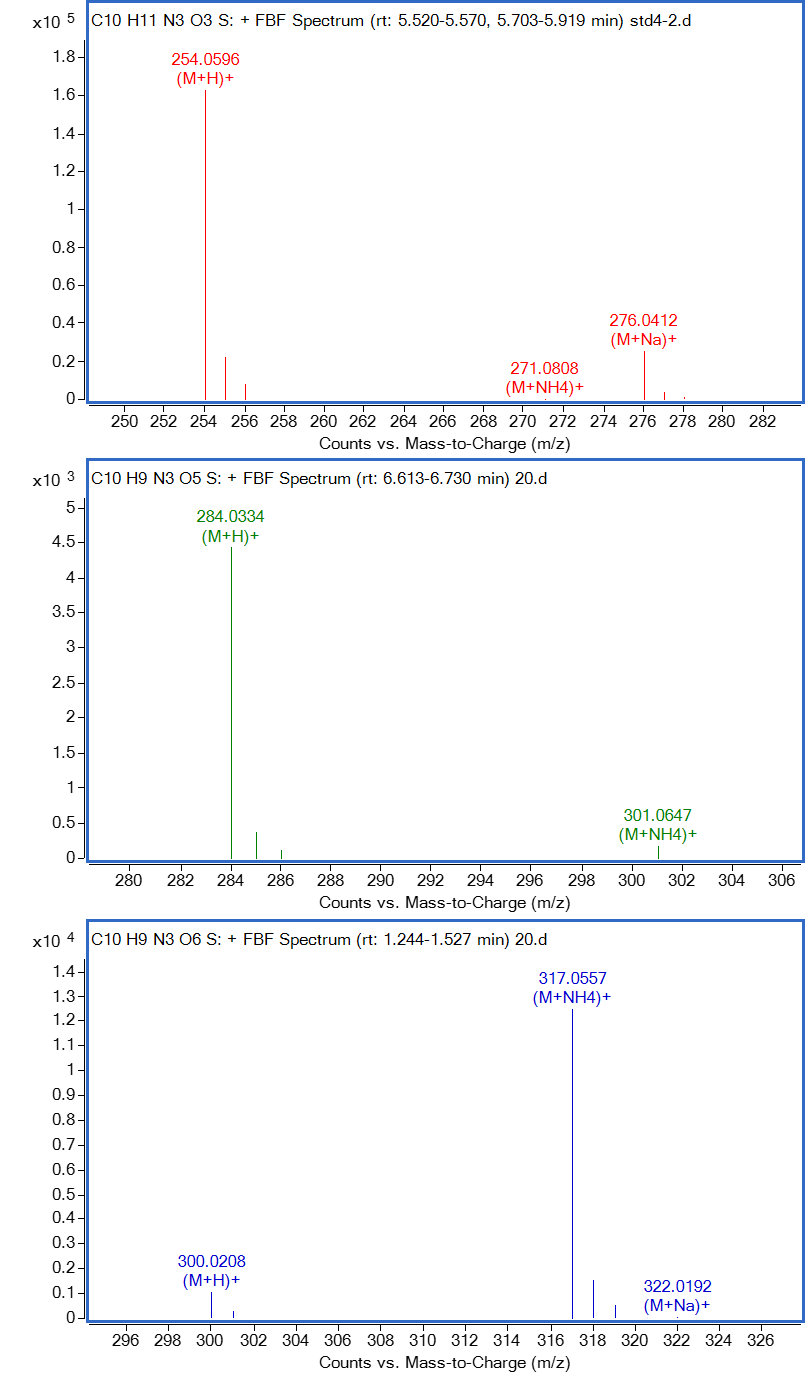


**Figure S5** Mass spectra of SMX and its intermediates.
